# Genetic diversity and spatial structure of the Rufous‐throated Antbird (*Gymnopithys rufigula)*, an Amazonian obligate army‐ant follower

**DOI:** 10.1002/ece3.2880

**Published:** 2017-03-19

**Authors:** Juliana Menger, Klaus Henle, William E. Magnusson, Antonella Soro, Martin Husemann, Martin Schlegel

**Affiliations:** ^1^ UFZ ‐ Helmholtz Centre for Environmental ResearchDepartment of Conservation BiologyLeipzigGermany; ^2^ Faculty of Biosciences, Pharmacy and PsychologyUniversity of LeipzigLeipzigGermany; ^3^ INPA ‐ Coordenação de Pesquisa em BiodiversidadeInstituto Nacional de Pesquisas da AmazôniaManausBrazil; ^4^ German Centre for Integrative Biodiversity Research (iDiv), Halle‐Jena‐LeipzigLeipzigGermany; ^5^ General ZoologyInstitute of BiologyMartin‐Luther‐University Halle‐WittenbergHalleGermany; ^6^ Centrum für NaturkundeUniversity of HamburgHamburgGermany

**Keywords:** antbirds, control‐region mtDNA, dispersal, microsatellites, tropical forests

## Abstract

Amazonian understory antbirds are thought to be relatively sedentary and to have limited dispersal ability; they avoid crossing forest gaps, and even narrow roads through a forest may limit their territories. However, most evidence for sedentariness in antbirds comes from field observations and plot‐based recapture of adult individuals, which do not provide evidence for lack of genetic dispersal, as this often occurs through juveniles. In this study, we used microsatellite markers and mitochondrial control‐region sequences to investigate contemporary and infer historical patterns of genetic diversity and structure of the Rufous‐throated Antbird (*Gymnopithys rufigula*) within and between two large reserves in central Amazonia. Analyses based on microsatellites suggested two genetically distinct populations and asymmetrical gene flow between them. Within a population, we found a lack of genetic spatial autocorrelation, suggesting that genotypes are randomly distributed and that *G. rufigula* may disperse longer distances than expected for antbirds. Analyses based on mitochondrial sequences did not recover two clear genetic clusters corresponding to the two reserves and indicated the whole population of the Rufous‐throated Antbird in the region has been expanding over the last 50,000 years. Historical migration rates were low and symmetrical between the two reserves, but we found evidence for a recent unilateral increase in gene flow. Recent differentiation between individuals of the two reserves and a unilateral increase in gene flow suggest that recent urban expansion and habitat loss may be driving changes and threatening populations of Rufous‐throated Antbird in central Amazonia. As ecological traits and behavioral characteristics affect patterns of gene flow, comparative studies of other species with different behavior and ecological requirements will be necessary to better understand patterns of genetic dispersal and effects of urban expansion on Amazonian understory antbirds.

## 1 Introduction

In an increasingly human‐modified environment, the ability of an organism to disperse from one place to another is essential, not only for its own fitness, but also for population dynamics and genetic structuring. As the amount of genetic differentiation is inversely related to the dispersal of individuals, and hence to gene flow, estimating genetic structure among spatially isolated populations provides an indirect quantification of effective dispersal across a landscape (Powell et al., 2013). Therefore, understanding the consequences of limited dispersal for genetic structuring is a useful tool to predict population responses to environmental changes and, consequently, to manage those populations (Bowler & Benton, 2005).

Gene flow mediated by dispersal can help connect geographically isolated populations, decrease relatedness among individuals, and reduce inbreeding (Ronce, 2007). Despite the benefits of dispersal, individuals moving in a matrix of nonsuitable habitat face energetic costs and increased mortality risks, which can affect population persistence (Fahrig, 1998; Gruber & Henle, 2008). In heterogeneous landscapes, gene flow is reduced by an increase in the isolation of habitat patches. This isolation can be due either to geographic distance, to physical natural barriers, such as rivers, mountains, or non‐suitable vegetation types, or result from human‐induced barriers, such as roads and urbanized areas (Fahrig, 2007; Keller & Largiadèr, 2003; Khimoun et al., 2016; Knowles & Richards, 2005; Milá, Wayne, Fitze, & Smith, 2009; Ribera & Vogler, 2004). Therefore, gene flow will depend on the dispersal ability of the organisms, which is influenced by their ecological traits and behavior (Baguette & van Dyck, 2007; Bélisle, 2005; Burney & Brumfield, 2009; Henle, Davies, Kleyer, Margules, & Settele, 2004).

Tropical ornithologists have long suspected that Amazonian forest understory birds are poor dispersers, particularly insectivorous antbirds (Thamnophilidae; Zimmer & Isler, 2003). Indeed, most species are year‐round residents, territorial, and have distributions restricted by large rivers (Cracraft, 1985; Greenberg & Gradwohl, 1986; Stotz, Fitzpatrick, Parker‐Iii, & Moskovits, 1996). At local scales, several studies have shown that antbirds avoid crossing forest gaps, and even narrow roads through a forest may act as barriers, limiting bird territories and movements (Develey & Stouffer, 2001; Laurance, 2004; Lees & Peres, 2009; Stouffer & Bierregaard, 1995). However, such studies are based on field observations and on plot‐based resighting or recapture of adult individuals, which may underestimate longer‐distance dispersal occurring through juveniles, that is, natal dispersal (Woltmann, Sherry, & Kreiser, 2012).

Using genetic markers to measure gene flow can overcome the limitations of field observations, as patterns of genetic variation give information on dispersal and population connectivity at scales at which field measures are ineffective (Koenig, van Vuren, & Hooge, 1996). Several studies that have investigated the effects of forest fragmentation on population‐genetic structure of Neotropical forest birds indicate that antbirds have greater levels of genetic structuring than other understory birds (Barnett, Ruiz‐Gutierrez, Coulon, & Lovette, 2008; Bates, 2002; Brown, Ramey, Tamburini, & Gavin, 2004; Burney & Brumfield, 2009; Woltmann, Kreiser, & Sherry, 2012). However, current genetic structure may not be a result of human‐induced habitat fragmentation, but of natural isolation acting over historical timescales (Chiucchi & Gibbs, 2010). As such, estimating the extent to which current genetic structure is a result of contemporary or historical processes is important for effective population‐based management plans (Converse et al., 2015).

**Figure 1:**
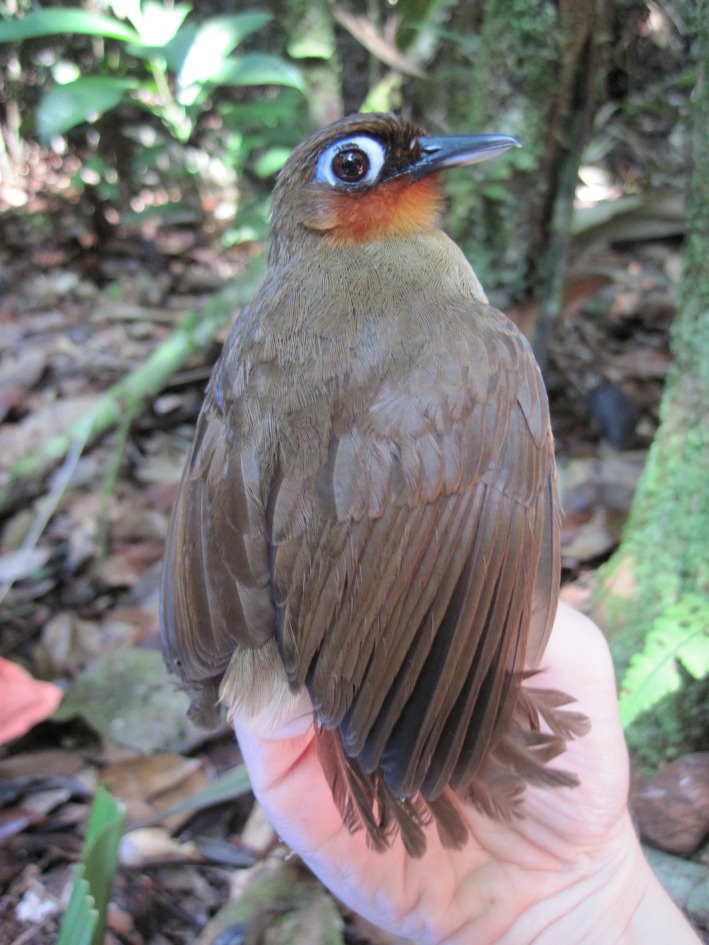
An individual of Rufous‐throated Antbird (*Gymnopithys rufigula*) captured at Ducke Forest Reserve, in central Amazonia. Photograph by J. Menger, August 2014

In this study, we used nuclear microsatellite markers and mitochondrial control‐region sequences to describe current and infer historical patterns of genetic structure of the Rufous‐throated Antbird (Thamnophilidae: *Gymnopithys rufigula*, Figure 1) in two large reserves north of the city of Manaus, Brazil. As the region surrounding the two reserves has been experiencing extensive urban development and construction and paving of roads over the last 40 years, we used a set of population‐genetic analyses to (1) test whether individuals sampled in the two reserves represent a single population or show signs of genetic differentiation; (2) estimate rates of migration between the two reserves; (3) estimate effective size of the local populations and assess recent changes in effective population size; and (4) reconstruct historical population‐size dynamics. As restricted dispersal over short distances is expected to generate fine‐scale spatial patterns of genetic structure, and sex‐biased dispersal may lead to differences in the genetic structure of males and females (Banks & Peakall, 2012; Peakall, Ruibal, & Lindenmayer, 2003), we additionally (5) investigated local genetic structure within a reserve and (6) sought differences in genetic structure between sexes.

## 2 Methods

### 2.1 Study species

The Rufous‐throated Antbird (*G. rufigula*: Thamnophilidae) is an obligate army‐ant‐following bird that inhabits the understory of Amazonian lowland *terra firme* forests (Ridgely & Tudor, 2009). It is a socially monogamous species that forms stable mated‐pair bonds (Zimmer & Isler, 2003). *Gymnopithys rufigula* breeds year round, with breeding peaks occurring between September and December (Johnson, Stouffer, & Bierregaard, 2012; Stouffer, Johnson, & Bierregaard, 2013). Normal clutch size is two eggs, and both parents brood and feed the chicks (Oniki, 1971). Rufous‐throated Antbirds maintain roosting and nesting territories; nesting pairs seldom feed more than 400 m away from their nests (Willis, 1967)**.** As a result of the nomadic behavior of the ants, however, Rufous‐throated Antbirds lack feeding territories and usually follow army‐ant swarms within large feeding ranges that often overlap neighboring mated pairs (Chaves‐Campos & Dewoody, 2008; O'Donnell, Logan, & Clayton, 2012). Nonetheless, long‐term monitoring of marked individuals indicates that the majority of movements of *G. rufigula* are within 1 km, but a few individuals are able to move distances greater than 10 km (van Houtan, Pimm, Halley, Bierregaard, & Lovejoy, 2007). Although medium‐sized (average mass based on 119 individuals = 28.6 g; *JM personal observation*), *G. rufigula* is dominant at ant swarms in central Amazonia, suggesting it has more access to resources provided by army‐ants than other ant‐following species and thus may need to move shorter distances than subordinate species (Repolho, 2012). Highly sensitive to forest fragmentation, the Rufous‐throated Antbird is among the first species to disappear from small forest patches (Harper, 1989; Stouffer & Bierregaard, 1995).

### 2.2 Study area

**Figure 2:**
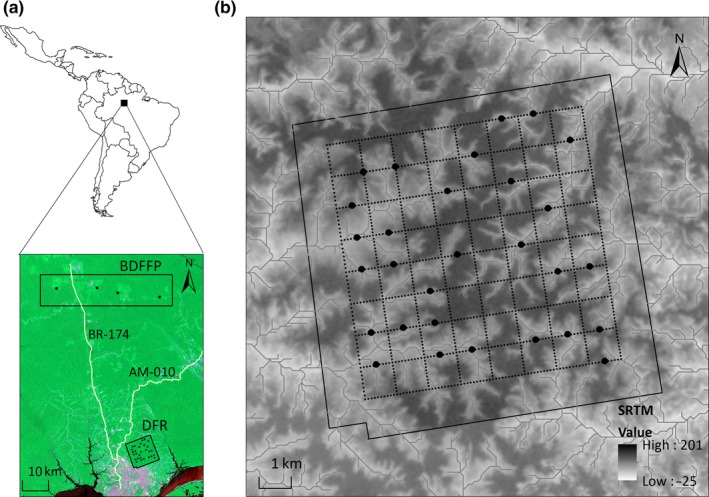
Map of the study area in central Amazonia, showing (a) the four sites at the Biological Dynamics of Forest Fragments Project (BDFFP, black dots inside rectangle) and sampling points at Ducke Forest Reserve (DFR, black dots inside square); (b) distribution of sampling points at DFR (black dots); topography, streams, and system of trails (dashed lines) are also shown

Genetic samples were obtained from two large reserves in central Amazonia: the Ducke Forest Reserve—DFR, located on the outskirts of the city of Manaus, Amazonas, Brazil (02°55′ – 03°01′S, 59°53′ – 59°59′W, Figure 2a), and the reserve of the Biological Dynamics of Forest Fragments Project—BDFFP, located 80 km north of Manaus (02°15′‐ 02°30′S, 59°40′‐ 60°05′W, Figure 2a). DFR is covered by 10,000 ha of old‐growth *terra firme* forest. Although the urban sprawl of Manaus has reached its southern and western limits, DFR is still connected to continuous forest on its eastern side and does not show any obvious impacts of urbanization within its limits. The BDFFP reserve is situated on three adjacent 15,000 ha ranches, covered by large expanses of old‐growth *terra firme* forests, but forest fragments and secondary forests are also present (Bierregaard Jr & Lovejoy, 1989; Cohn‐Haft, Whittaker, & Stouffer, 1997; Powell et al., 2015). The DFR and the BDFFP are roughly 50 km apart from each other, and, although large stretches of old‐growth forests between them still persist, the highways BR‐174 and AM‐010 and several small roads disconnect the two reserves. The construction of the two main roads began in the late 1970s, but paving was completed only in the late 1990s (Rodrigues & Pinheiro, 2011). Since then, the area deforested in the Manaus region associated with these roads has increased 200%, mainly due to urban growth (Rodrigues & Pinheiro, 2011).

### 2.3 Sampling collection

We sampled 80 individuals (40 males and 40 females) of *G. rufigula* in DFR during the dry season of two consecutive years (2012–2013). Birds were captured with mist nets (9 m long, 32 mm mesh size) at 29 sampling points systematically placed at intervals of roughly 1 km (Figure 2b). The number of individuals per sampling point ranged from one to eight (see Table S1.1). Rufous‐throated Antbirds exhibit little sexual dimorphism, but once captured, males and females can be readily distinguished by their interscapular patch, which is white in males and tawny‐orange in females. Each individual was banded, and a blood sample of approximately 50 μl was collected via brachial venipuncture and stored in absolute ethanol or in Queen's lysis buffer (Seutin, White, & Boag, 1991). Birds were released unharmed. All activities involving birds were conducted under approval of the Brazilian Center for Bird Conservation—CEMAVE (Permit 3576), and the Brazilian Biodiversity Authorization and Information System—SISBIO (Permit 34850). Blood samples were deposited in the Genetic Resource Collection of the Instituto Nacional de Pesquisas da Amazônia—GRC‐INPA.

We also obtained 40 blood vouchers of *G. rufigula* (20 males and 20 females) that were collected by other researchers between 2010 and 2011 in four locations within BDFFP (Figure 2a) and made available through the GRC‐INPA. The number of samples per location ranged from seven to 14 (see Table S1.1). Because exact geographic coordinates of the individual samples from BDFFP were not available, our within‐population spatial analyses were restricted to DFR.

### 2.4 Microsatellite genotyping

DNA was extracted from blood samples using the Wizard Genomic DNA Purification Kit (Promega, Madison, USA), following the manufacturer's protocols. Individuals of *G. rufigula* were genotyped at 14 microsatellite loci described in Menger, Gerth, Unrein, Henle, and Schlegel (2017) using protocols and PCR conditions therein. PCR products were run on an Applied Biosystems 3130xl Genetic Analyzer using GeneScan 500 ROX size standard by the Interdisziplinäres Zentrum für klinische Forschung (IZKF) of the University of Leipzig (Leipzig, Germany). Size scoring was performed with Peak Scanner Software v.1.0 (Applied Biosystems, Foster City, USA). The presence of null alleles was checked with Micro‐Checker v.2.2.3 (van Oosterhout, Hutchinson, Wills, & Shipley, 2004).

### 2.5 Genetic diversity and genetic differentiation

We calculated the number of alleles per locus, observed and expected heterozygosities (*H*_o_ and *H*_e_), deviations from Hardy–Weinberg equilibrium (HWE), and performed exact tests of linkage disequilibrium between pairs of loci for DFR and BDFFP using Genepop Web v.4.2 (Rousset, 2008). Allelic richness was estimated using the rarefaction method implemented in the Hierfstat package (Goudet & Jombart, 2015) in R v.3.2.5 (R Core Team, 2016).

We calculated pairwise genetic differentiation *F*_ST_ (Weir & Cockerham, 1984) between all 29 sampling points within DFR and between all four locations within BDFFP separately. As we did not find significant genetic differentiation within none of the reserves (see Tables S1.2 and S2), we pooled the data and calculated genetic differentiation *F*_ST_ between DFR and BDFFP. All tests were calculated in FSTAT v.2.9.3.2, using permutations to obtain significance (Goudet, 2001).

### 2.6 Population inference

We employed two Bayesian clustering approaches to infer the number of genetically distinct populations (*K*) among the sampled sites. Firstly, we used Structure v.2.3.4 (Pritchard, Stephens, & Donnelly, 2000), assuming an admixture model with correlated allele frequencies and the LOCPRIOR model (Hubisz, Falush, Stephens, & Pritchard, 2009). To identify the best estimate of *K* from 1 to 8, we set a burn‐in period of 50,000 followed by additional 150,000 iterations; 20 replicates were run at each *K*. We determined *K* based on the log posterior probability of the data for a given *K* (Pritchard et al., 2000) and on the rate of change in the log probability of the data between successive clusters—the Δ*K* statistic (Evanno, Regnaut, & Goudet, 2005). These analyses were performed in Structure Harvester v.0.6.94 (Earl & Vonholdt, 2011). We finally averaged all 20 runs at the best *K* with Clumpp v.1.1.2 (Jakobsson & Rosenberg, 2007) and visualized the results with Distruct v.1.1 (Rosenberg, 2004).

Secondly, we used the spatially explicit cluster model implemented by the Geneland v. 4.0.5 package (Guillot, Mortier, & Estoup, 2005) in R (R Core Team, 2016). In a first step, we set the Markov chain Monte Carlo (MCMC) at 100,000 iterations with a burn‐in period of 1,000. The number of genetic units (*K*) to be tested was set between 1 and 8 with a correlated allelic frequency model. To assess the consistency of the results, we ran the MCMC 10 times and chose the best result based on the highest average posterior probability. In a second step, we ran 100 simulations using the same parameter settings as described above, with *K* fixed to the number of clusters inferred therein, to assign individuals to the clusters.

### 2.7 Migration estimates

To estimate recent migration rates between sites, we used BayesAss v.3.0 (Wilson & Rannala, 2003). BayesAss uses a Bayesian approach and MCMC sampling to estimate migration (*m*) over the last few generations (Wilson & Rannala, 2003). Following Chiucchi and Gibbs (2010) and Converse et al. (2015), we assumed that meant roughly five generations. We ran BayesAss with 10 million iterations, a sampling frequency of 2,000, a burn‐in of 10%, and otherwise default settings.

We also estimated historical migration rates with Migrate‐N v.3.6 (Beerli, 2009). Under a coalescent framework, Migrate‐N estimates migration rates (measured as mutation‐scaled immigration rate M) up to ~4Ne generations (thousands of years). To approximate the stepwise‐mutation model for microsatellites, we ran Migrate‐N under a Brownian‐motion model. We used slice sampling to run four statically heated parallel chains (heated at 1.0, 1.5, 3.0, and 1,000,000) for 1,000,000 iterations, sampled every 100,000 iterations, and excluded 1,000 iterations as burn‐in. MCMC estimates of *m* were modeled with prior boundaries of 0 and 500,000 (lower and upper bounds, respectively). We used a full migration model and considered parameter estimates accurate when an effective sample size (ESS) >1,000 was observed (Converse et al., 2015).

To be able to compare migration estimations resulted from Migrate‐N (M = *m*/μ) to those resulting from BayesAss (*m*), we multiplied M values by a commonly assumed microsatellite mutation rate of 5 × 10^−4^ (Chiucchi & Gibbs, 2010; Converse et al., 2015).

### 2.8 Effective population size

We estimated the effective size (*N*_e_) of the inferred populations based on the linkage disequilibrium method corrected for sample size bias, using the monogamous mating model and lowest allele frequency = 0.050, as implemented in NeEstimator v.2.01 (Do et al., 2014). We also pooled individuals of the two reserves to calculate an overall *N*_e_, using the same settings as described above. To test for recent changes in the effective population size, we used the software Bottleneck v.1.2.02 (Peery et al., 2012). In recently bottlenecked populations, a heterozygosity excess relative to the number of alleles present in the population is expected, while a deficit is expected in the case of a population expansion (Cornuet & Luikart, 1996; Rieux, De Lapeyre De Bellaire, Zapater, Ravigne, & Carlier, 2013). We performed the analysis with 9,999 iterations under the assumptions of the stepwise‐mutation model (SMM) and the two‐phase mutation model (TPM; variance = 20, proportion of SMM = 95%).

### 2.9 Spatial autocorrelation within DFR

To investigate fine‐scale patterns of genetic structure within DFR, we conducted spatial autocorrelation analysis in GeneAlEx (Peakall et al., 2003; Smouse & Peakall, 1999). We used a pairwise geographic and a pairwise squared genetic‐distance matrix to calculate the spatial autocorrelation coefficient *r* and tested statistical significance with 9,999 random permutations and 9,999 bootstrap estimates (Peakall et al., 2003; Smouse & Peakall, 1999). We calculated *r* for two different distance classes: 400 m and 1 km. We chose these distance classes based on the maximum distances that nesting obligate army‐ant‐following birds may wander (400 m; Willis, 1967), and on the minimum distance between sampling sites (1 km). If a positive significant genetic structure was present, we expected *r* to decrease as the distance class increases.

### 2.10 Sex‐biased dispersal

We calculated four statistics commonly used to assess the overall extent of sex‐biased dispersal, as implemented in Hierfstat package (Goudet & Jombart, 2015) in R (R Core Team, 2016). To do so, we pooled the data from the two reserves and calculated for each sex the mean corrected assignment index (mAIc), the variance of the corrected assignment index (vAIc), *F*_IS_, and *F*_ST_ (Goudet, Perrin, & Waser, 2002). Under sex‐biased dispersal, the most dispersive sex is expected to have lower *F*_ST_ and mAIc values, but higher *F*_IS_ and vAIc values (Goudet et al., 2002). Significance of *t*‐values was estimated using 9,999 permutations.

Additionally, we used spatial autocorrelation analyses to test whether different dispersal by males and females results in differences in spatial structure between sexes within DFR (Banks & Peakall, 2012). Firstly, we conducted the analysis separately for males and females, using distance classes of 400 m and 1 km. Then, we compared the patterns of genetic structure between sexes by determining the 95% bootstrap confidence intervals (CI) for the autocorrelation *r* values for each sex (Banks & Peakall, 2012). If differences in dispersal existed, we expected CIs between sexes not to overlap, with *r* values significantly greater in the dispersal‐restricted sex.

### 2.11 Mitochondrial control‐region DNA sequencing

We amplified a fragment of the mitochondrial control‐region DNA using primers particularly designed for this study (Fwd: 5′‐CCACATCACATCCACGCCAAAAAG‐3′; Rev: 5′‐GTGTTGATGGATACGTGTAAGAAGATG‐3′). PCR amplifications were performed in 25 μl total volume containing 16.8 μl double‐distilled water, 1 μl genomic DNA (10–20 ng/μl), 0.2 μl DreamTaq Green DNA polymerase, 5 U/μl and 2.5 μl DreamTaq Green buffer 10× (Thermo Fisher, Schwerte, Germany), 2.5 μl dNTPs (2 mmol/L), 1 μl forward primer (10 mmol/L), and 1 μl reverse primer (10 mmol/L). Thermal cycling proceeded as follows: 95°C for 5 min; followed by 30 cycles of 95°C for 45 s, 58°C for 45 s, and 72°C for 60 s; finishing with 72°C for 2 min. PCR products were cleaned using NucleoSpin Gel and PCR Clean‐up Kit (Macherey‐Nagel, Düren, Germany). Sequencing was carried out on an Applied Biosystems ABI 3730 XL by GATC Biotech (Cologne, Germany). We used BioEdit v.7.2.5 to visualize, edit, and align DNA sequences (Hall, 1999). We used default settings to obtain a ClustalW (Thompson, Higgins, & Gibson, 1994) alignment of the sequences and then trimmed its edges to equal sequence lengths.

### 2.12 Mitochondrial diversity and haplotype network

Nucleotide (Pi) diversity and haplotype (Hd) diversity for each population were estimated using DnaSP v.5.10.01 (Librado & Rozas, 2009). To visualize relationships among haplotypes, we constructed a minimum spanning network (MSN, Clement, Posada, & Crandall, 2000) using Popart (Leigh & Bryant, 2015).

### 2.13 Historical demography

We determined the most suitable substitution model for the whole population based on the Bayesian information criterion (BIC) with jModelTest2 v.2.1.10 (Darriba, Taboada, Doallo, & Posada, 2012). The best substitution model achieved by jModelTest2 was HKY + invariable sites. The HKY is a nucleotide substitution model that assumes every base has a different equilibrium base frequency and that transitions and transversions evolve at different rates (Hasegawa, Kishino, & Yano, 1985). We then reconstructed historical population‐size dynamics using the Bayesian coalescent skyline plot method (Drummond, Rambaut, Shapiro, & Pybus, 2005) as implemented in Beast v.1.8.2 (Drummond, Suchard, Xie, & Rambaut, 2012). We performed the analysis using the substitution model chosen by jModelTest2 under a strict‐clock model and the general avian substitution rate of mitochondrial evolution of 2.1% sequence divergence per million years (Lovette, 2004; Weir & Schluter, 2008). Runs of 100 million steps were performed, sampling every 10,000 steps under default settings. Skyline plots were constructed using Tracer v.1.6 (Rambaut, Suchard, Xie, & Drummond, 2014).

## 3 Results

### 3.1 Genetic diversity and genetic differentiation

We scored 120 individuals at 14 loci. One locus (Gyru05) showed evidence for null alleles and was therefore removed from subsequent analyses (Table 1). There was no indication of HWE departure in any site. No pair of loci was found to be in linkage disequilibrium. DFR had a mean number of alleles of 12.4, ranging from 3 to 21, while the mean number of alleles for BDFFP was 11.9 and ranged from 2 to 21 (Table 1). From a total of 179 alleles observed, 16 were private to DFR and 23 to BDFFP. Observed heterozygosity and allelic richness did not differ between sites (Table 1). We detected a weak, but significant genetic differentiation between DFR and BDFFP (*F*_ST_ = 0.009, *p *<* *0.000).

**Table 1:** Number of alleles (*N*_A_), allelic richness (*A*_R_), observed (*H*_o_) and expected (*H*_e_) heterozygosity, and Wright's fixation index (*F*_IS_) of 14 microsatellite loci within populations of the Rufous‐throated Antbird in central Amazonia. Values in bold indicate significant departure from HWE, after Bonferroni correction. Gyru05 was not included in further analyses, as it showed evidence of null alleles

	DRF					BDFFP				
	*N*_A_	*A*_R_	*H*_o_	*H*_e_	*F*_IS_	*N*_A_	*A*_R_	*H*_o_	*H*_e_	*F*_IS_
Gyru02	21	19.13	0.875	0.921	0.050	21	20.79	0.900	0.942	0.046
Gyru03	21	19.41	0.925	0.913	−0.014	20	19.86	1.000	0.921	−0.087
Gyru05	11	10.71	0.638	0.819	**0.222**	12	11.32	0.525	0.806	**0.352**
Gyru06	16	12.37	0.763	0.774	0.015	17	13.59	0.875	0.838	−0.044
Gyru07	20	18.49	0.863	0.913	0.055	20	19.97	0.850	0.918	0.075
Gyru10	15	13.12	0.900	0.899	−0.002	15	13.99	0.850	0.913	0.069
Gyru11	19	17.07	0.900	0.917	0.019	18	18.33	0.925	0.934	0.009
Gyru12	10	9.25	0.663	0.777	0.148	10	9.55	0.800	0.809	0.011
MyEx26	5	4.90	0.413	0.414	0.004	5	4.93	0.450	0.527	0.148
CAM17	7	6.47	0.725	0.711	−0.019	4	6.18	0.525	0.566	0.073
CAM18	5	4.69	0.425	0.479	0.113	5	4.62	0.525	0.571	0.081
TG01‐040	3	2.94	0.138	0.153	0.102	3	3.13	0.075	0.074	−0.017
TG02‐088	17	15.28	0.975	0.919	−0.061	15	15.47	0.950	0.910	−0.045
TG12‐015	3	2.50	0.225	0.284	0.209	2	2.33	0.250	0.222	−0.130
All loci (mean)	12.2	11.17	0.673	0.706	0.048	11.93	11.72	0.679	0.711	0.046

DFR, Ducke Forest Reserve; BDFFP, Biological Dynamics of Forest Fragments.

### 3.2 Population inference

**Figure 3:**
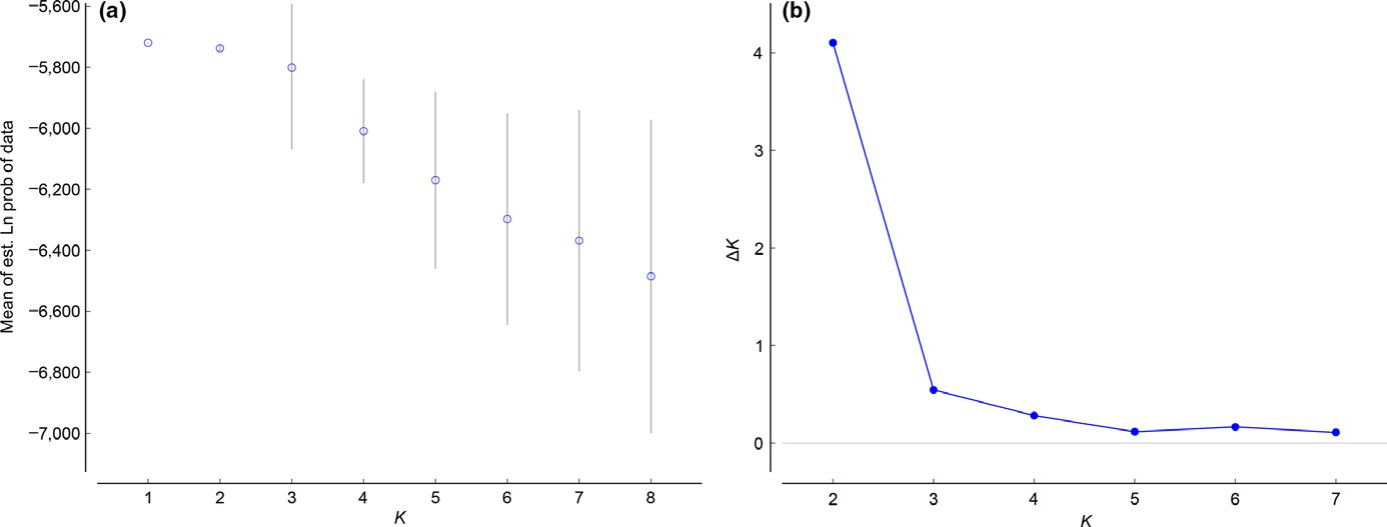
Number of populations inferred by Structure. a) Mean log likelihoods for each *K* (±*SD*), and the rate of change in the log probability of the data between successive clusters (Δ*K*)

**Figure 4:**
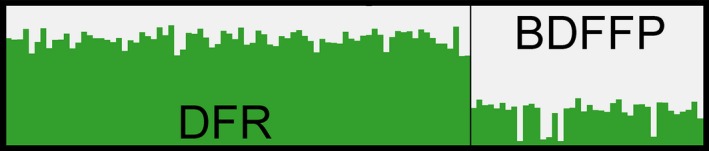
Population structure of *Gymnopithys rufigula* estimated from Structure for *K *=* *2 genetic clusters. Each column represents an individual whose estimated membership to either cluster is indicated by the two different colors. DFR, Ducke Forest reserve; BDFFP, Biological Dynamics of Forest Fragments Project

**Figure 5:**
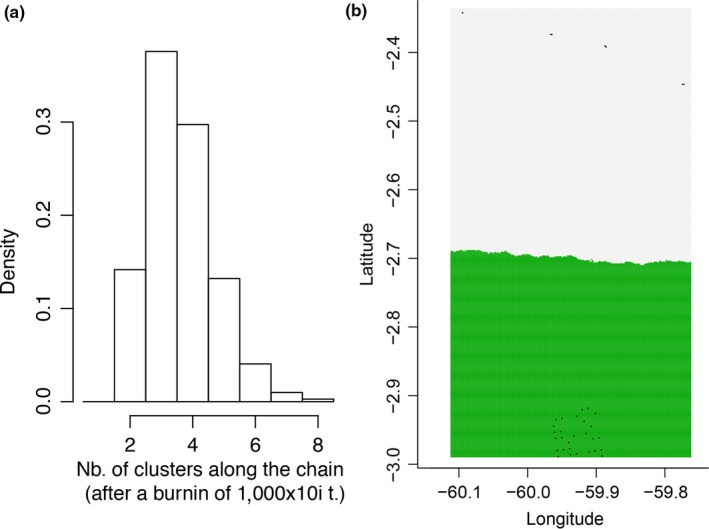
Number of populations inferred by Geneland: (a) Highest mean posterior density supports *K *=* *3; (b) assignment of individuals to the clusters supports *K *=* *2. Colors indicate the estimated cluster membership, dots represent sampling points of DFR and sampling locations of BDFFP

The highest log posterior probability of the data and the highest value for Δ*K* obtained via Structure analysis suggested *K *=* *2 (Figure 3). We hence assigned membership of each individual to the clusters with *K* set to 2. All individuals were assigned to their most likely genetic cluster with a threshold >0.6 (Figure 4). The number of genetic clusters estimated by Geneland was *K *=* *3 in eight of 10 runs. In the remaining two runs, *K *=* *2 was obtained. The run with the highest mean posterior density likewise had a *K *=* *3 (Figure 5a). Thus, to assign individuals to clusters, the final simulation was performed with *K* set to 3. The assignment step, however, led to only two populations (Figure 5b), showing that the third inferred population was not modal for any of the individuals; hence, no individual was assigned to it. Such “ghost populations” are interpreted as spurious artifacts that have not been successfully removed by the MCMC algorithm (Guillot, Estoup, Mortier, & Cosson, 2005). Thus, the final two genetic clusters inferred by Geneland corresponded to DFR and BDFFP populations, as also suggested by the Structure analysis.

### 3.3 Migration estimates

Estimates of contemporary migration obtained from BayesAss suggested high self‐recruitment rates for both populations (DFR = 0.99 [±0.01], BDFFP = 0.68 [±0.02]) with asymmetrical gene flow between DFR and BDFFP: higher rates of dispersal occurred from DFR to BDFFP (0.32 [±0.02]) than the opposite (0.01 [±0.01]). Estimates of historical migration obtained from Migrate‐N indicated little and symmetrical gene flow between the two reserves: migration from DFR to BDFFP was = 0.013 [±0.004], and from BDFFP to DFR was = 0.007 [±0.004]. These results also suggest a recent increase in migration rates from DFR to BDFFP, but no overall change in migration rates from BDFFP to DFR.

### 3.4 Effective population size

Harmonic‐mean estimates revealed an effective population size of 517 individuals (CIs = 302–1,455) for DFR and 170 individuals for BDFFP (CIs = 118–287). The estimated overall *N*_e_ was 1,189 individuals (CIs = 588–16,970). Both populations showed evidence of heterozygosity deficiency, indicating a recent expansion in their effective population size (Wilcoxon's signed‐rank SMM tests; DFR: *p *= 0.001; BDFFP: *p *=0.047). However, when a TPM model was used, the multilocus probability of displaying a deficiency in heterozygosity appeared to be significant only for DFR (Wilcoxon's signed‐rank TPM tests; DFR: *p *=0.039; BDFFP *p *=0.190).

### 3.5 Spatial autocorrelation within DFR

**Figure 6:**
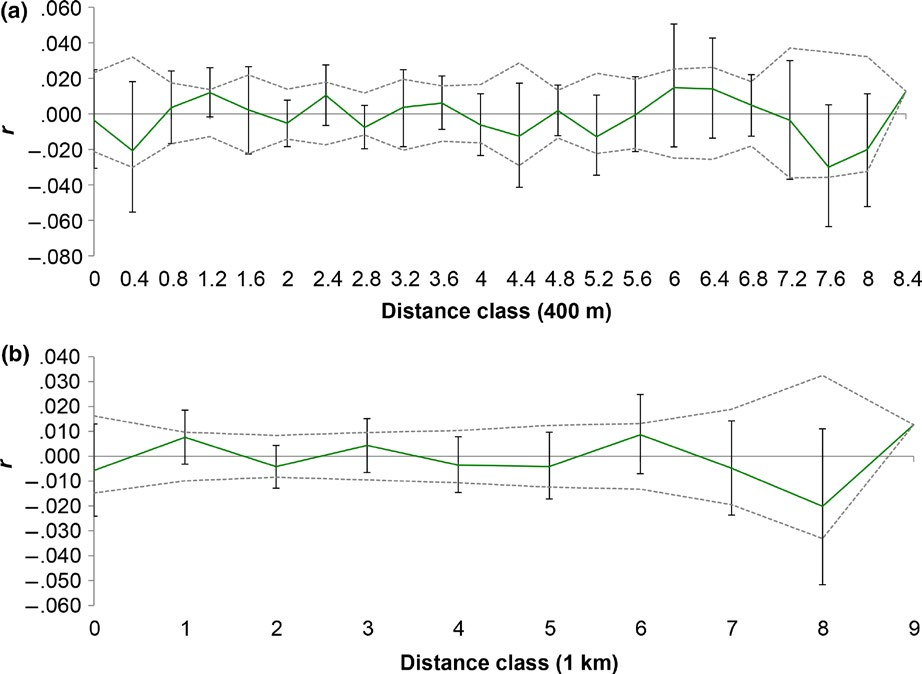
Correlogram of the genetic autocorrelation coefficient *r* as a function of distance generated from 80 individuals within the Ducke Forest Reserve (DFR). The 95% bootstrapped (error bars) and permuted (dashed lines) confidence intervals are shown for distance classes of 400 m (a) and 1 km (b)

Both correlograms (400/m and 1/km distance classes) showed that the autocorrelation coefficient *r* fell inside the 95% permuted confidence intervals, and bars of 95% bootstrapped estimates crossed zero at all distance classes, indicating no statistically significant spatial autocorrelation within DFR (Figure 6).

### 3.6 Sex‐biased dispersal

**Figure 7:**
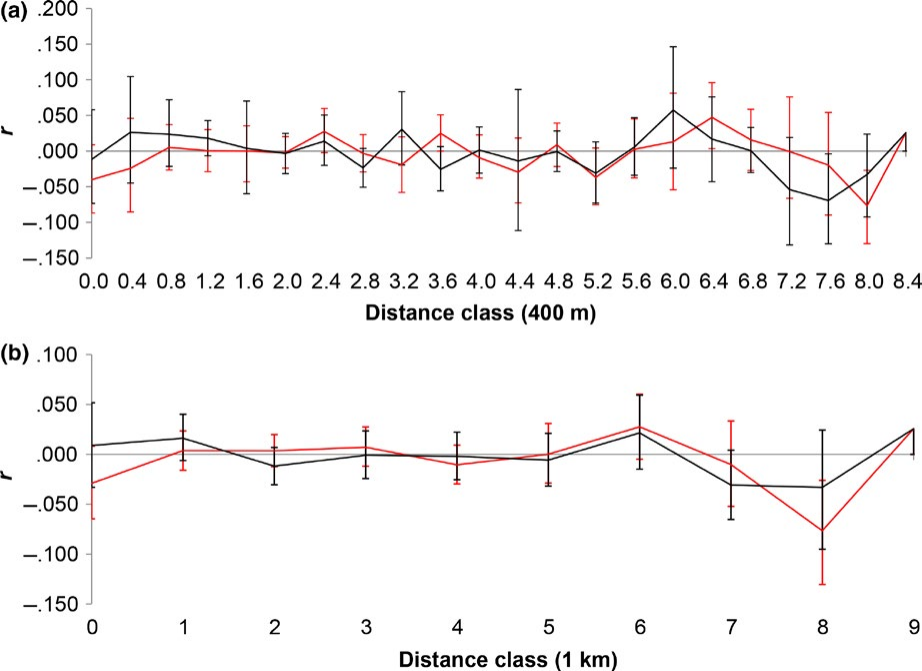
Correlogram comparing the genetic autocorrelation coefficient *r* between males (black line) and females (red line) as a function of distance within Ducke Forest Reserve (DFR). The 95% bootstrapped (error bars) confidence intervals are shown for distance classes of 400 m (a) and 1 km (b)

Analyses of sex‐biased dispersal indicated no overall differences in dispersal by males and females. There was no significant difference in mAIc (*t *=* *0.133, *p *=* *0.899), vAIc (*t *=* *0.766, *p *=* *0.876), or *F*_ST_ (*t *=* *−0.007, *p *=* *0.246) between sexes. However, *F*_IS_ values were slightly greater for males (*t *=* *−0.051, *p *=* *0.036). Within DFR, the correlograms comparing spatial autocorrelation between sexes showed that *r* values for males and females overlapped in both distance classes (Figure 7).

### 3.7 Mitochondrial diversity and haplotype network

**Figure 8:**
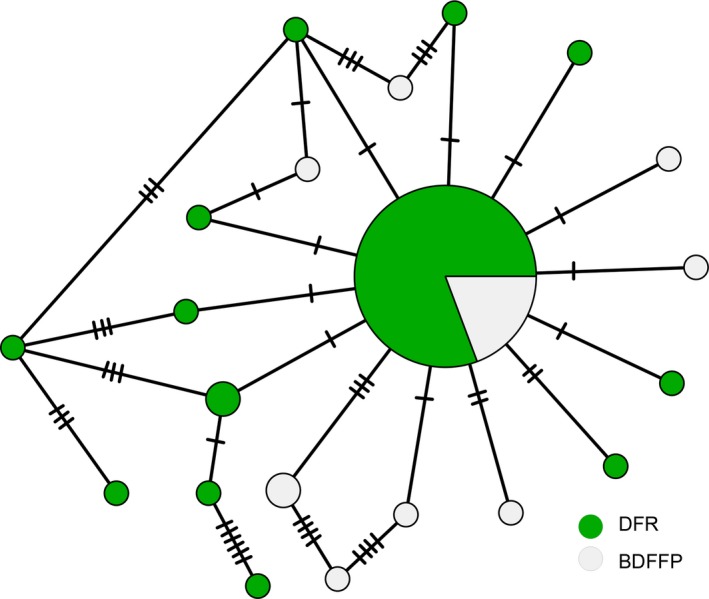
Estimates of relationships among haplotypes for Rufous‐throated Antbird based on minimum spanning network analysis (MSN) of 79 mitochondrial control‐region DNA sequences. The size of circles indicates the number of individuals, and colors represent the two sites—Ducke Forest Reserve (DFR) and Biological Dynamics of Forest Fragments (BDFFP)

We successfully amplified 79 mitochondrial control‐region DNA sequences of ~ 700 bp (DFR = 59, BDFFP = 20). We found 21 haplotypes within the 79 sequences of *G. rufigula*. Overall haplotype diversity was 0.481 and nucleotide diversity was 0.002. Nucleotide diversity did not differ between sites (DFR Pi = 0.001, BDFFP Pi = 0.003), but BDFFP had higher haplotype diversity (DFR Hd = 0.395, BDFFP Hd = 0.705). Only the most frequent haplotype was shared between the two populations; 12 were found only in DFR, and eight were recorded only in BDFFP (Figure 8).

The haplotype network did not reflect a separation into two distinct groups. Rather, it reflected a star‐like topology with a shared haplotype between the two reserves in the center, and rare variants radiating from the ancestral sequence, a pattern expected after range or demographic expansions (Figure 8).

### 3.8 Historical demography

**Figure 9:**
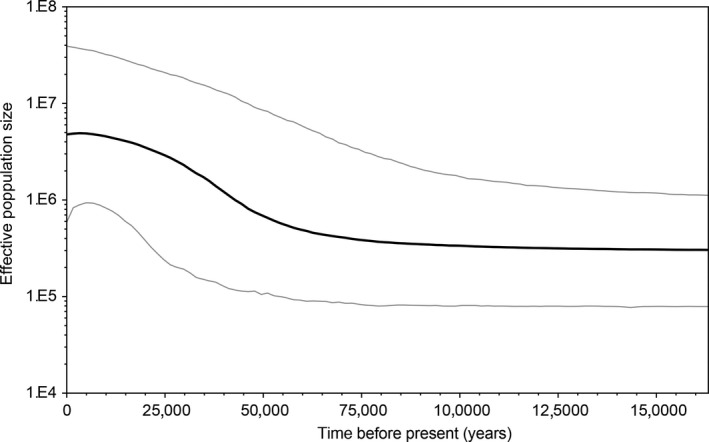
Bayesian skyline plots based on ~700 bp of 79 mitochondrial control‐region sequences showing the demographic history of the whole Rufous‐throated Antbird population in the study area. The black line represents the median value, while the gray lines indicate the 95% Bayesian credibility intervals

Analyses based on mitochondrial control‐region DNA sequences suggested demographic expansion for the whole Rufous‐throated Antbird population. Bayesian skyline plot estimates showed a signal of population expansion over the last 50,000 years (Figure 9).

## 4 Discussion

This is the first study to describe the population‐genetic structure of the Rufous‐throated Antbird. Currently, individuals of *G. rufigula* in the Manaus region form two distinct genetic clusters, which are undergoing an expansion in effective population size. Although the star‐like shape of the haplotype network indicates that both reserves shared an ancestral population whose structure cannot be distinguished from a panmictic population in expansion, the presence of haplotypes unique to each reserve suggests that the two areas have been undergoing a process of differentiation and that anthropogenic changes are likely to be driving recent changes in population dynamics.

### 4.1 The presence of two genetic clusters

Although our results suggest the presence of two genetic clusters, these might just be segments of the same population that are isolated by distance. Nonetheless, the four sites within BDFFP are distributed over a maximum distance of ~ 40 km, which is roughly the same distance as between the DFR and BDFFP reserves (~50 km, for all pairwise geographic distances, see Tables S1.2 and S2), but formed a single apparently panmictic population. Apart from the westernmost, the other sites within BDFFP are connected by large extensions of old‐growth forests allowing the dispersal of individuals. However, it is important to note that each site within the BDFFP has a relatively small sample size (between seven and 14 genotypes). Because sample size is reported to affect the ability of Structure in assigning individuals (Evanno et al., 2005), the apparent lack of genetic differentiation among BDFFP sites may be due to inadequate sampling.

On the other hand, the two main roads (BR‐174 and AM‐010) that run between DFR and BDFFP, as well as the urban sprawl of the city of Manaus, may be contributing to genetic differentiation between these local populations. Several studies have reported road avoidance by a wide range of vertebrates and showed that roads affect population‐genetic structure, not only by reducing the abundance of individuals and gene flow among populations, but also by increasing mortality risks (Balkenhol & Waits, 2009; Jackson & Fahrig, 2011). Moreover, a recent multispecies study showed that forest specialists are more reluctant to cross urbanized areas, leading to genetic divergence between populations (Khimoun et al., 2016). As such, those roads may act as barriers to dispersal and lead to population‐genetic differentiation through gene‐flow limitation. However, an effective test supporting statistically the effect of roads in the dispersion/migration of the Rufous‐throated Antbird would only be possible with more individuals sampled in each location, as well as larger number of replicates on either side of the road.

### 4.2 Recent asymmetry in migration rates

Our results indicate incongruence between historical and contemporary estimates of migration. Interestingly, we found low and symmetrical migration rates from BDFFP to DFR over the long term, but a recent increase in the proportion of individuals immigrating from DFR into BDFFP. While current gene flow may be a continuation of past patterns, recent anthropogenic activities are likely to be augmenting this effect and causing a unilateral increase in gene flow.

It has been reported that extinction‐prone Neotropical birds had a reduction in long‐distance movement after forest fragmentation (van Houtan et al., 2007). Moreover, Afrotropical forest‐dependent birds also showed a depression in migration rates after habitat fragmentation (Callens et al., 2011) and similar fragmentation effects are well known for a range of animal taxa (e.g., Hoehn, Sarre, & Henle, 2007). If Rufous‐throated Antbirds are able to disperse longer distances in large areas of forest, but avoid crossing forest gaps, individuals moving from BDFFP to DFR may display a boundary response when encountering a road (Fahrig, 2007) and stay in their most suitable habitats. While BDFFP reserve is linked to close‐by enormous areas of continuous forest, the DFR has been pressured by the urban sprawl of Manaus on its western and southern sides. As such, individuals that leave the reserve are possibly forced to use the eastern and northern sides where more suitable environments are found. Even though some individuals may avoid crossing roads, others are still able to cross them and effectively contribute to gene flow between the two reserves, as suggested by our migration analysis. Indeed, it is likely that other understory antbirds may be experiencing the same phenomenon, particularly solitary species.

### 4.3 Population size and expansion

Estimates of current effective population size revealed that both reserves are under the effective population‐size limit of 1,000 individuals considered critical to maintain evolutionary potential in a long‐term perspective (Frankham, Bradshaw, & Brook, 2014). However, there was evidence for a recent expansion in effective population size in both reserves, in agreement with historical patterns. Nonetheless, it should be noted that estimators of recent changes in effective population size rely on the assumptions that the population is at mutation–drift equilibrium, without immigration or population substructure and, therefore, may not be consistent with data from natural populations (Cornuet & Luikart, 1996).

### 4.4 Lack of fine genetic structure within DFR

We found an apparently random distribution of genotypes within DFR. This suggests that Rufous‐throated Antbirds may regularly disperse distances over 10 km. Our results confirm patterns of long‐term capture–recapture data that some individuals are able to disperse longer distances (van Houtan et al., 2007) and are in agreement with results for another army‐ant follower, the Ocellated Antbird *Phaenostictus mcleannani* in Costa Rica, where the spatial distribution of genotypes also approached randomness (Chaves‐Campos & Dewoody, 2008). These patterns are likely to be explained by the dependence of obligate ant‐following birds on swarms of army‐ants. Army‐ant followers obtain their food by catching arthropods and small vertebrates flushed from the leaf litter by raiding ant swarms, especially *Eciton burchellii* (Willis & Oniki, 1978). Because *bivouacs* (living nests) of *E. burchellii* are nomadic and widely spaced, obligate army‐ant‐following birds have developed a series of ecological and behavioral adaptations to effectively exploit army‐ant swarms as a food resource (O'Donnell et al., 2012); accordingly, most army‐ant followers perform *bivouac* checking and lack feeding territories (Chaves‐Campos & Dewoody, 2008; Swartz, 2001; Willis, 1967; Willson, 2004). As a result, obligate army‐ant followers may range widely when tracking *bivouacs*, move outside their roosting/nesting territories, and even cross other territories (O'Donnell et al., 2012). While the distribution of *G. rufigula* at fine scales may be related to the position and movements of *E. burchellii*, it also could be explained by their roosting/nesting territoriality: mated pairs in their own territories dominate trespassers (Willis, 1967). Thus, juveniles of territorial, long‐lived species such as antbirds have to disperse longer distances in search of vacant territories to establish. As such, small territories of the adults, combined with their long life spans could result in juveniles having to disperse large distances between hatching and establishing territories.

### 4.5 Sex‐biased dispersal

Although female‐biased dispersal is expected for birds (Clarke, Sæther, & Røskaft, 1997; Greenwood, 1980), our results indicate that male and female *G. rufigula* disperse similarly. Other studies have also found little evidence of sex‐biased dispersal in Neotropical understory birds (Chaves‐Campos & Dewoody, 2008; Woltmann, Kreiser, et al., 2012). Like most antbirds, *G. rufigula* maintains stable mated‐pair bonds and parental care is shared by both males and females (Oniki, 1971). Under a monogamic mating system, as seen in *G. rufigula*, males and females will disperse equally, as they share the same costs of parental care and dispersal; that is, both sexes are subjected to the same competition processes and have same variance in reproductive success (Brom, Massot, Legendre, & Laloi, 2016). Nonetheless, these results need to be interpreted with caution, as differences in dispersal between males and females need to be intense in order to be detected using microsatellite data (Goudet et al., 2002), and the power of detecting sex‐biased dispersal in spatial autocorrelation analysis might also be affected by the sample size (Banks & Peakall, 2012).

### 4.6 Private haplotypes

The presence of haplotypes unique to each reserve indicates low current levels of genetic exchange between DFR and BDFFP, but might also suggest adaptation to local environments, such as climate conditions and food resources (Sjöstrand, Sjödin, & Jakobsson, 2014). For instance, some studies have shown morphological adaptations of the flight apparatus in birds occupying forest fragments (Anciães & Marini, 2000; Hermes, Döpper, Schaefer, & Segelbacher, 2016). It is possible that Rufous‐throated Antbirds occupying DFR (and even other understory birds) have adaptations to enhance mobility and flight capacity. However, as we do not have morphological data to test this idea, further research that combines genetic and morphological data is needed to elucidate how private haplotypes and local adaptations are linked.

### 4.7 Historical demography

Both the haplotype network and the reconstruction of historical population‐size dynamics identified signs of population expansion, starting ~ 50,000 years before present, indicating that the Rufous‐throated Antbird population expanded throughout the Last Glacial Maximum (LGM): 19,000–26,000 years ago (Clark et al., 2009). Thus, our results support the hypothesis that glacial‐age forests were similar to modern lowland *terra firme* forests (Colinvaux, de Oliveira, Moreno, Miller, & Bush, 1996) or that the expansion of suitable climatic conditions during the LGM could have allowed Neotropical forests and forest‐dwelling species to expand (Leite et al., 2016).

### 4.8 Conservation implications

Although its global population size has not been quantified, *G. rufigula* has a large distributional range and is thus considered as of least concern by the IUCN Red List (BirdLife International, 2016). However, it has been shown that IUCN does not efficiently account for geospatial data, which could shrink the distributional ranges of many species and increase the number of species that need to be considered at risk (Ocampo‐Peñuela, Jenkins, Vijay, Li, & Pimm, 2016). This could be the case of *G. rufigula*, which is confined only to the Guiana Shield, northern Amazonia (Naka, Bechtoldt, Henriques, & Brumfield, 2012). As such, the small effective population size in these two reserves coupled with the high sensitivity to forest fragmentation displayed by the species (van Houtan, Pimm, Bierregaard, Lovejoy, & Stouffer, 2006) indicates that these local populations of Rufous‐throated Antbirds may be prone to environmental stochasticity and thus might not be viable in the long term.

Given that the urbanization of the region around the two reserves may be driving genetic erosion in the population of Rufous‐throated Antbirds, more studies are needed to understand how environmental changes induced by human activities, particularly roads, affect Amazonian biodiversity. Moreover, taking into account that the Brazilian Amazon is rapidly undergoing extensive development and that 17.000 km of new roads are added every year to its road network (Ahmed, Souza, Riberio, & Ewers, 2013), evaluation of the effects of these roads is urgent. Road planning that includes routing, corridors, over and underpasses, and bridges rather than landfill to cross valleys could facilitate dispersal and gene flow between otherwise isolated populations.

Comparative studies of other Amazonian antbirds and sedentary understory species will be necessary to better understand patterns of genetic dispersal of Amazonian birds, given that ecological traits and behavioral characteristics affect gene flow and responses of species to fragmentation (Bregman, Sekercioglu, & Tobias, 2014; Henle et al., 2004). As such, studies of other bird species are essential to identify which species are most likely to be negatively affected by limited gene flow due to urbanization and road construction and to increase connectivity between populations. Studies encompassing a greater number of sites, species, individuals, and spatial scales are necessary to evaluate the generality of our findings.

## Conflict of Interest

None declared.
