# Supplementary material for: Genetic diversity and spatial structure of the Rufous‐throated Antbird (Gymnopithys rufigula), an Amazonian obligate army‐ant follower
Source: Ecol Evol. 2017 Mar 19;7(8):2671–84. doi: 10.1002/ece3.2880 (PMC5395437; doi:10.1002/ece3.2880)
Supplement: Supplementary file 1 [file ECE3-7-2671-s001.docx]

**Supporting Information S1**

**Table S1.1.** Number of individuals sampled in each of the 29 *sampling points* within DFR and in each of the four *locations* within BDFFP.

| Sampling point / location | Number of individuals |
| --- | --- |
| DFR – LO1_5500 | 1 |
| DFR – LO1_6500 | 1 |
| DFR – LO2_1000 | 3 |
| DFR – LO2_2000 | 1 |
| DFR – LO2_4500 | 4 |
| DFR – LO2_7500 | 2 |
| DFR – LO3_0500 | 4 |
| DFR – LO3_3500 | 1 |
| DFR – LO3_5500 | 5 |
| DFR – LO4_0500 | 1 |
| DFR – LO4_1500 | 2 |
| DFR – LO4_6500 | 4 |
| DFR – LO5_0500 | 3 |
| DFR – LO5_1500 | 2 |
| DFR – LO5_3500 | 2 |
| DFR – LO5_5500 | 4 |
| DFR – LO6_2500 | 4 |
| DFR – LO6_6500 | 1 |
| DFR – LO6_7500 | 3 |
| DFR – LO7_0500 | 3 |
| DFR – LO7_1500 | 1 |
| DFR – LO7_2500 | 2 |
| DFR – LO8_0500 | 3 |
| DFR – LO8_2500 | 2 |
| DFR – LO8_3500 | 2 |
| DFR – LO8_5500 | 2 |
| DFR – LO8_6500 | 8 |
| DFR – LO8_7500 | 2 |
| DFR – LO9_7500 | 7 |
| BDFFP – west | 12 |
| BDFFP – mid-west | 7 |
| BDFFP – mid-east | 7 |
| BDFFP – east | 14 |

**Table S1.2.** F_ST_ values between pairs of locations within BDFFP (lower diagonal) and their respective pairwise geographic distances (in meters, upper diagonal). Indicative adjusted P-value for multiple comparisons was 0.008. No pairwise comparison was found to be significant after 120 permutations.

|  | BDFFP east | BDFFP mid-east | BDFFP mid-west | BDFFP west |
| --- | --- | --- | --- | --- |
| BDFFP east | 0 | 14967 | 22803 | 39181 |
| BDFFP mid-east | -0.0014 | 0 | 8100 | 24160 |
| BDFFP mid-west | 0.0203 | -0.0046 | 0 | 16223 |
| BDFFP west | 0.0299 | 0.0150 | 0.0078 | 0 |
